# Improving Protection to Prevent Bacterial Infections: Preliminary Applications of Reverse Vaccinology against the Main Cystic Fibrosis Pathogens

**DOI:** 10.3390/vaccines11071221

**Published:** 2023-07-09

**Authors:** Mario Cocorullo, Laurent R. Chiarelli, Giovanni Stelitano

**Affiliations:** Department of Biology and Biotechnology “Lazzaro Spallanzani”, University of Pavia, Via A. Ferrata 9, 27100 Pavia, Italy; mario.cocorullo01@universitadipavia.it (M.C.); laurent.chiarelli@unipv.it (L.R.C.)

**Keywords:** reverse vaccinology, bacterial infection, cystic fibrosis

## Abstract

Reverse vaccinology is a powerful tool that was recently used to develop vaccines starting from a pathogen genome. Some bacterial infections have the necessity to be prevented then treated. For example, individuals with chronic pulmonary diseases, such as Cystic Fibrosis, are prone to develop infections and biofilms in the thick mucus that covers their lungs, mainly caused by *Burkholderia cepacia* complex, *Haemophilus influenzae*, *Mycobacterium abscessus complex*, *Pseudomonas aeruginosa* and *Staphylococcus aureus.* These infections are complicated to treat and prevention remains the best strategy. Despite the availability of vaccines against some strains of those pathogens, it is necessary to improve the immunization of people with Cystic Fibrosis against all of them. An effective approach is to develop a broad-spectrum vaccine to utilize proteins that are well conserved across different species. In this context, reverse vaccinology, a method based on computational analysis of the genome of various microorganisms, appears as one of the most promising tools for the identification of putative targets for broad-spectrum vaccine development. This review provides an overview of the vaccines that are under development by reverse vaccinology against the aforementioned pathogens, as well as the progress made so far.

## 1. Introduction

Cystic fibrosis (CF) is a rare genetic autosomal recessive disease that affects mainly the respiratory tract, but also many exocrine organs (pancreas, hepatobiliary system, exocrine glands), intestine and reproductive tract [1,2]. Mutations on the CFTR gene, that is located on chromosome 7, are indeed causative of an altered function of the relative chloride channel protein, with the consequential production of viscous mucus that damages the secretion ducts and, finally, the organs [1].

Patients with Cystic Fibrosis (pwCF) have an average life span of around 44 years and often die from lung failure [1]. Moreover, they mostly have complications caused by infections in the respiratory tract, since many bacteria may colonize the thick mucus mentioned above [2]. Among all the pathogens, five species are commonly found in infected lungs of pwCF: the most common pathogens infecting young pwCF are *S. aureus* (SA) and *H. influenzae*, while as patients age, they are mainly colonized by one or a few bacteria including *Pseudomonas aeruginosa* (PA)*, Burkholderia cepacia* complex (Bcc), and *Mycobacterium abscessus complex* (Mab) [2,3,4,5]. Those bacteria are often hard to eradicate because of their natural resistance to antibiotics due to several factors, including a thick wall that may prevent a drug to enter the cell, detoxifying enzymes that may inactivate the drug or efflux pumps that may extrude a drug outside the cell before reaching its target [2]. Moreover, bacteria may develop biofilms, communities of the same or different species, impenetrable to most drugs [2]. Finally, bacteria may acquire new antimicrobial resistances through spontaneous mutation or horizontal gene transfer that could occur both intra- and inter-species [6].

A major improvement in CF therapy has resulted from the introduction of CTFR modulators, small molecule drugs capable of restoring defective channel function, with significant benefits for the health and quality of life of patients [7]. However, modulators are not suitable for all CTFR mutations, and due to the high cost of these drugs, they are not accessible in most parts of the world. For these reasons, new therapeutic approaches are still required, especially with regard to infections.

Indeed, the actual therapeutic regimens based on antibiotics are not enough to fight the pathogens in pwCF and alternative therapies are fine-tuning in support. Among these approaches, the administration of nitric oxide represents a promising antimicrobial strategy, particularly against Mab [2]. Another interesting approach is the phage therapy, the use of bacteriophages to fight specific infections, which has recently been considered again and successfully for the treatment of pwCF [2,8]. Clearly, many efforts still are being made to develop more effective drugs that may hit unexplored targets [8], specifically, new anti-virulence compounds such as quorum sensing inhibitors [8,9]. Indeed, this strategy does not aim to kill the pathogens but rather to prevent the attack on the host and would not exert a selective pressure, thus reducing the risk of resistance [9]. Of course, another important strategy to prevent infections in pwCF is the development of novel preventive vaccines [10].

Vaccines have importantly changed and improved throughout time. Starting from the cross-species vaccination, widely used in Asia during the 12th century and introduced in Europe by Jenner in 1796 with the cowpox vaccine to induce immunity to smallpox [11], safer and continuously effective vaccines are now available, including protein, antigen-based or DNA and RNA vaccines [12]. Vaccine development has also changed deeply in recent years as well. For example, some epitope-based vaccines have been developed by structural vaccinology through the engineering of a protein structure to improve its antigenic surface, such as the influenza virus haemagglutinin (HA) vaccine [13]. 

One of the most recent strategies of vaccine development is based on identification of putative antigenic proteins through a genetic approach, a strategy named reverse vaccinology [10]. This review focuses on providing an overview about the vaccines that are in development against the above described, most important pathogens of pwCF through reverse vaccinology: a powerful prevention tool to improve health and the lifestyle of those people.

## 2. Reverse Vaccinology on the Last Decade of Strategies for Vaccine Development

Reverse vaccinology (RV) is a genome-based approach for vaccine development that differs to the traditional methods of vaccine design, which utilizes killed or attenuated pathogens such as the measles vaccine [14]. Indeed, the term “reverse” refers to the identification of specific genes among the whole pathogen genome to be selected and expressed as recombinant proteins for the implementation of the vaccine. Anyway, among all the proteins, only specific epitopes can be recognized by the host immune system [15,16] and their identification is one the main problems and limitations in vaccine development. In this sense, RV integrates immunological information to rationally select the most attractive antigens for further studies [17]. In this way, it is possible to select proteins that efficiently stimulate the immune response: an essential prerequisite for the development of effective vaccines [18].

Traditional approaches have limitations that are bypassed by RV. For example, the number of identified antigens, usually limited with biochemical methods, is overcome by RV while taking into account every putative antigen during the preliminary analysis. Another example is the antigen variability among strains and species that RV is able to reduce by selecting the most conserved genome sequences [17]. Finally, costs and speed of vaccine production need to be considered during the design, since they are important limitations for vaccine development and distribution, for example, in the case of an emergency, as evidenced by the COVID-19 outbreak.

Notably, RV is a time-saving approach; it avoids the necessity to grow the pathogens for the analysis in exchange of an often faster production of recombinant proteins. Finally, modern high-throughput computational analysis tools allow the development of powerful vaccines with different antigens against one pathogen, but also broad-spectrum vaccines that exploit the same antigen against different pathogens [18,19,20].

Nevertheless, reverse vaccinology, as well as structural vaccinology, have some limitations. Among these, RV cannot identify carbohydrate-based antigens, which are common protective antigens of pathogens. Another important drawback of RV is that it depends on the genomes of the analyzed organisms and on the software (and equations) utilized. Despite the fact that the second issue can be overcome by using multiple software, or by integrating and refining the goodness of the system, the necessity of well sequenced whole genomes of different strains of the target microorganism still remains.

Indeed, the advancements in bioinformatics and structural biology, along with a deeper knowledge of the genomes, contributed to the development of this approach in the past few years. Several software and webtools have been developed in order to identify the best antigens for vaccines; the first was Vaxign in 2010, up to the most recent VaxiJen, Jenner-Predict, NERVE and VacSol. However, the main limit of this strategy still lies in the computational methods, such as the research equations used to identify the perfect antigen, which are indeed constantly under implementation [19,20].

**Figure 1:**
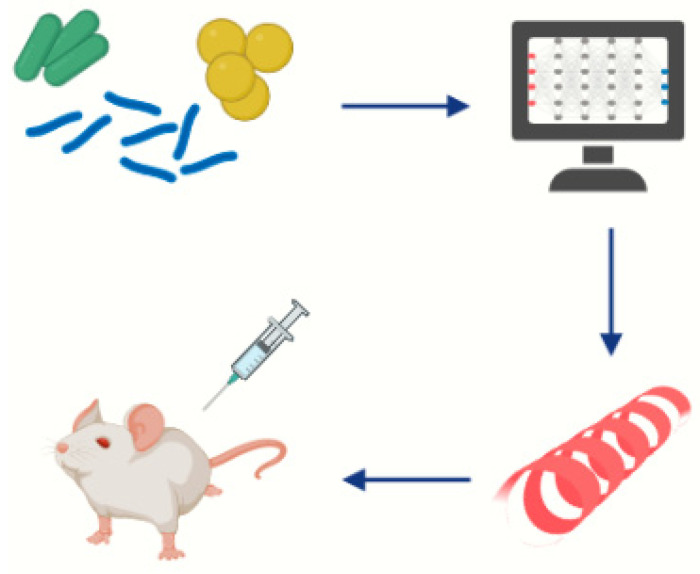
Schematic representation of vaccine development through Reverse Vaccinology approach, from the choice of the pathogen to the pre-clinical evaluation.

Once the potential antigen is identified, the corresponding gene is cloned in an expression vector and the recombinant protein is expressed and further evaluated in vitro and in vivo for the immune response, for example, by ELISA, and finally in animal models before reaching clinical studies (Figure 1) [21]. This validation is essential, not only to confirm the effectiveness of the selected antigens but also to prove its safety for an autoimmune response and its suitability as a vaccine candidate.

One of the well known examples of RV is the first vaccine developed with this strategy, the MenB vaccine, which has become a model for vaccine design and opened a new path to realize powerful tools against pathogenic bacteria. The MenB vaccine targets four different antigens of the serogroup B strain of *Neisseria meningitidis*: the factor H-binding protein (FHbp), the neisserial adhesin A (NadA), the neisserial heparin-binding antigen (NHba), and the outer membrane vesicle (OMV) [14,22]. This vaccine has been a great improvement for quality of life, since it provides protection against most, but not all, pathogenic *N. meningitidis* strains [23,24,25]. 

Different vaccines have been developed with RV since MenB commercialization, and many studies are ongoing against pathogenic bacteria. For example, the classical BCG vaccine against *M. tuberculosis* has shown a low efficacy in the population, and for this reason, it is mandatory to search for new strategies, along with the development of new drugs [26,27]. Several vaccines against this pathogen are under development, some of which are in clinical trial. Additionally, ongoing studies are attempting to expand the possibility to develop an effective vaccine against tuberculosis through RV [28,29,30].

## 3. Vaccine against *Burkholderia cepacia* Complex

The *Burkholderia cepacia* complex (Bcc) encompasses more than 20 closely related species, including those most commonly found in pwCF *B. cenocepacia* and *B. multivorans* [31]. Although the incidence of Bcc infections in pwCF is relatively low, about 3%, they are commonly associated with a severe decline in lung function, which can develop into a systemic infection called epacian syndrome [32]. Moreover, Bcc members, and in particular *B. cenocepacia*, has a remarkable adaptability to environmental changes and are naturally resistant to several antibiotics commonly used in clinical practice, making their treatment very challenging.

Therefore, the development of a vaccine could represent an important approach to prevent Bcc infections in pwCF, although relatively few studies have been conducted so far [33]. 

However, although numerous efforts have been made to develop vaccines against Bcc species, with several studies reporting the possibility to achieve partial immunoprotection, so far, no candidates have progressed to clinical trials [33].

Nevertheless, in vivo vaccination studies in mice have reported some degrees of protection against Bcc using different approaches. For instance, differently formulated preparations of *B. multivorans* outer membrane proteins were able to prevent early colonization of *B. multivorans* and *B. cenocepacia*, ameliorating lung damage [34,35]. 

Another study used the proteomics approach to isolate proteins potentially involved in the attachment of bacteria to lung epithelial cells, identifying seven proteins including the adhesins linocin and OmpW [3]. Immunization of mice with these two proteins produced in recombinant form was found to elicit a potent humoral response, which protected against both *B. cenocepacia* or *B. multivorans* [36].

By contrast, live attenuated cells or heat-killed cells vaccines have not been extensively tested against Bcc [37], although this approach has been successfully assayed in mice models against *B. mallei* or *B. pseudomallei* [38]. One of these studies showed that the disruption of the tonB gene, involved in iron transport, significantly reduced the virulence of *B. mallei* [39]. Based on this evidence, Pradenas and colleagues developed a tonB mutant *B. cenocepacia* strain. This strain, which proved unable of actively transporting iron, effectively protected mice against *B. cenocepacia* challenge, upon intranasal administration [37]. 

More recently, several approaches have been used to select vaccine candidates, including immunoproteomic techniques. For instance, Sousa and co-workers characterized the immunoproteome of a *B. cenocepacia* strain using serum samples from cystic fibrosis patients [40]. This work identified seven membrane-associated proteins that showed a positive reaction with the pool of CF patients’ serum samples. However, several other proteins that were found to be immunoreactive cannot be identified using the two-dimensional electrophoresis. This issue has been also found in previous works [41]. Indeed, the surface exposed bacterial proteins, which represent the most appealing antigens, are usually difficult to study due to their hydrophobicity and low water solubility, as well as their low abundance [42]. To solve this issue, the same authors optimized the so-called “surface-shaving” methodology for Bcc bacteria [43]. This approach consists of digesting live, intact cells with proteases, to recover surface-exposed peptides and proteins that can be then analyzed by liquid chromatography and mass spectrometry. The main advantages of this method over classical proteomics are that it is a gel-free approach, thus overcoming the problems of electrophoretic analysis of membrane proteins. Moreover, shaving allows to identify proteins that are more accessible to proteases, and thus to the host immune system [44]. In this work, among 263 potentially surface-exposed proteins, 16 have been experimentally identified by shaving, and three of them proved to be immunogenic, confirming the suitability of this approach for the development of vaccines against Bcc [43].

More recently, reverse vaccinology has also been considered to identify potential vaccine targets in *Burkholderia* species. Actually, most of these works have been performed on *B. pseudomallei* and are mainly limited to in silico studies [45,46,47]. However, some investigations against Bcc are also emerging. For instance, Alsowayeh and colleagues used a reverse vaccinology and immunoinformatic approach to predict effective antigens for the design of a multi-epitope chimeric vaccine; an in silico model was predicted to have strong interactions with host immune receptors [48]. Although only in silico studies have been performed at present, they should nevertheless provide a solid foundation for the future development of new vaccine candidates against *B. cepacia* complex.

## 4. *Haemophilus influenzae* Vaccines, Not Only against Meningitis

*Haemophilus influenzae* is a Gram-negative pathobiont that may cause opportunistic infection in the airway mucosal surface [49]. It can be divided into six capsulated serotypes, from a to f, and other non-capsulated strains named non-typable or NTHi [50]. Against the most virulent strain, the type b (Hib), which mainly causes meningitis, an effective vaccine is available and has been commonly introduced in the vaccination schedule of many countries. The Hib vaccine is classified as protein-conjugated polysaccharide, since it is composed by a carrier protein linked to polyribosyl ribitol phosphate (PRP), which is part of the Hib polysaccharide capsule [50]. Actually, four proteins from different pathogens are conjugated to PRP to cover a wide range protection: a nontoxic mutant *Corynebacterium diphtheriae* protein CRM197 (PRP-CRM), the Neisseria meningitidis outer membrane complex (PRP-OMP), the tetanus toxoid (PRP-TT) and the diphtheria toxoid (PRP-D) [50]. Anyway, pwCF needs complete protection against *H. influenzae* serotypes that are not covered by the Hib vaccine, but, also, against NTHi, which may play a role in biofilm formation, as reported in the literature by many case-study and research articles [51,52].

So far, only one case of RV is present in the literature about the development of novel vaccines against *H. influenzae*. Indeed, using the ReVac bioinformatic tool, a pool of putative candidates for a novel vaccine have been selected, such as the Hemopexin transporter and the outer membrane lipoprotein P4 [52]. Both candidates have been selected starting from a pool of 270 NTHi genomes derived from sputum isolates of adult patients with chronic pulmonary diseases. Among the 477 769 predicted proteins, the best candidates have been selected through specific filters to be highly conserved among the species, so that the putative vaccine may cover a wide range of strains [52]. Sadly, the main objective of this work is just theorical, namely, to validate the ReVac tool for vaccine development, while the experimental validation of results is not planned, at least for the Hemopexin transporter protein. The lipoprotein P4, instead, has already been largely studied over the past 30 years as a putative vaccine candidate antigen against NTHi *H. influenzae* strains [53,54].

## 5. Non-Tuberculous Mycobacteria Vaccines

Non-tuberculous mycobacteria (NTM) encompass more than 30 species able to cause infections in humans, some of which are increasing in incidence in pwCF, causing a significant increment in morbidity and mortality [55]. The most commonly identified NTM in pwCF belongs to the *Mycobacterium avium* complex (MAC), which includes *Mycobacterium avium* subsp. *avium* (Mav) and *Mycobacterium avium* subsp. *intracellulare*, and to the Mycobacterium abscessus complex (MABSC), which encompasses the *M. abscessus* subspecies *abscessus* (Mab), *bolletii* (*M. bolletii*), and *massiliense* (*M. massiliense*) [56]. 

Among NTMs, Mab is becoming the pathogen of most concern in CF centers, with a standard treatment that involves a multidrug therapy consisting of at least three antibiotics for up to two years, resulting in several issues for the patients, a low probability of success, and a significant increase in drug resistance [56]. Therefore, there is an urgent need of novel strategies to improve the prevention of NTM infections among pwCF, including the development of effective vaccines. 

Actually, several vaccine candidates against NTM, and in particular, against Mab, have been reported, but no vaccine is currently available or in clinical development [57]. 

However, the antitubercular vaccine Bacillus Calmette Guerin (BCG), still used to prevent serious forms of tuberculosis in children and adolescents in hyperendemic countries, has been shown to provide cross-protection against *M. avium* and *M. abscessus* infection. For this reason, its use as a prophylactic or a therapeutic vaccine against NTM has been suggested [58]. Nevertheless, several *M. abscessus* antigens have been investigated for the development of specific vaccines. For instance, Le Moigne et al. identified the phospholipase PLC as a valid target, developing a DNA-based vaccine candidate, able to rapidly clear *M. abscessus* in vaccinated mice. However, this vaccination induced a high response against the smooth variant of Mab, but not against a highly virulent and rough variant [59]. Subsequently, the same researchers developed a DNA vaccine based on the virulence factor MgtC, which was assayed in a mouse model of cystic fibrosis. The formulated vaccine led to the high production of an antibody against MgtC, as well as a rapid decrease in bacterial burden in vaccinated mice [60]. Interestingly, a possible immune cross-reactivity with other CF pathogens, such as *P. aeruginosa* and *B. cenocepacia*, have been hypothesized for both PLC and MgtC vaccines as both of these microorganisms express these virulence factors [60]. Moreover, the same group developed a cell-based bioassay to isolate the components of the *M. abscessus* cell wall that mostly stimulates the Toll-like receptor 2 (TLR2)-mediated inflammation. These extracts, consisting mainly of lipoproteins and proteins were named TLR2eF and administered to a mouse model of cystic fibrosis. Interestingly, TLR2eF administration in a nebulized form resulted in low protection against Mab, while intravenous administration led to high-antibody production [61].

The growing interest in NTM, particularly Mab, led to the disclosure of several complete genomes from different strains, further expanding the possibility of identifying vaccine candidates through reverse vaccinology [62]. For instance, Cornejo-Granados et al. used a bioinformatics pipeline to predict the secretome of the ATCC 19977 *M. abscessus* strain and of 15 clinical isolates, providing interesting support for the systematic investigation of candidate proteins for the development of vaccines [63]. In another work, Dar and colleagues collected 34 genome sequences of Mab from the NCBI GenBank database, in order to perform a pangenome-reverse vaccinology study with the view to design a multi-epitope vaccine, with very promising in silico data [64]. However, the majority of these studies are limited to in silico investigations, and still need to be experimentally confirmed. Nevertheless, they represent a valuable starting point for the identification of potential epitopes to be thoroughly investigated for the development of effective candidate vaccines against *M. abscessus*.

## 6. *Pseudomonas aeruginosa*

*Pseudomonas aeruginosa* (PA), one of the most studied bacteria, is a Gram-negative opportunistic pathogen, which can cause either acute or chronic infections, notably in pwCF. PA is classified as multi-drug resistant (MDR), characterized by a number of virulence factors and an adaptable genome, which will make the microorganism able to overlap the ongoing treatments. For this reason, the research for new antibiotics and antivirulence compounds is pursued simultaneously with that for vaccines against *P. aeruginosa* [65,66,67]. Vaccine targets include factors that trigger the immune response in the host, characterized by antigenic elements typically found on the bacterial surface, such as lipopolysaccharides (LPS) and outer membrane proteins (OMPs) [68]. In addition, inner membrane proteins have also been evaluated and further classified by the amount of the most prevalent amino acid and their physical-chemical features [67]. RV has been largely exploited over the genome of *Pseudomonas aeruginosa* PAO1 strain, evaluating elements such as lectins and glycan-binding proteins that are involved in adherence to host cells, in order to carry out the bacterial colonization and the formation of the biofilm [69,70]. Other putative vaccines obtained by RV are represented by the chaperone-usher pathway, penicillin binding protein of the bacterial cell wall, extracellular type III secretory system, antibiotic efflux pump, etc. [71]. 

Since iron is essential for Gram-negative bacteria to establish infections, iron acquisition proteins (IAPs) are attractive targets for vaccine development, including against *P. aeruginosa* [65]. Bioinformatics analysis allowed us to choose the best target, taking into account many features such as frequency of expression, antigenicity and solubility, which are important for the subsequent building of recombinant proteins. Moreover, the immunogenic protein must be different from every human protein to avoid autoimmunity and, ideally, must be present in all the strains of the bacterium. The development of a multi-epitope vaccine can start from these assumptions, merging many epitopes from different IAPs. For instance, HasAP, PhuR and HxuA, which are involved in the iron scavenging from porphyrins have been evaluated as the target for the development of new vaccines [65]. 

What makes the extensive in silico screening effective, is that it provides unknown antigens in addition to the known ones, confirming the reliability of this method. In this way, many proteins have been identified, many of which belong to outer membrane proteins, which are the first line of virulence effects. Furthermore, by combining the different immunogenic elements found by bioinformatics analysis, many in vivo experiments yielded acceptable outcomes, in particular, merging the protein PA5340, which seems to exist only in *P. aeruginosa*. Subsequently, a chimeric multi-epitope vaccine has been developed, starting from the different computational analysis, but also using linker and adjuvant to increase the response of the immune system [66,67,68]. In bacteria such as *P. aeruginosa*, the most interesting targets include proteins involved in virulence, which are essential for adhesion, mobility and the penetration in the host cells. The computational analysis allowed the identification of putative targets, which can be both secreted and surface-exposed proteins, and are involved in many processes such as resistance mechanisms and pathogenicity. Moreover, in silico analysis uncovered many epitopes from porin proteins located in the outer membrane, but also from uncharacterized proteins. These results have been evaluated by IC_50_ analysis, discarding the less attractive proteins and filtering out only the most powerful epitopes. This analysis revealed two unknown proteins, namely PA1288 and PA4874, characterized by many features, which make them interesting in the research of new target for the development of vaccines, including their presence in the lungs of pwCF, thus confirming their involvement in *P. aeruginosa* infection. This example shows how the development of multi-epitope vaccines is facilitated by RV, filtering out all the unlikely proteins for this purpose. Vaccine efficacy may be enhanced by building chimeric vaccines. Bioinformatic analysis further confirmed that these kinds of vaccines are effective and highly stable [67,68,69]. 

Putative vaccines have also been evaluated among proteins that bind to the host glycans: a common cell adhesion strategy among bacteria [70]. In particular, studies identified *P. aeruginosa* lectins, which bind with glycans in pwCF as well as immunocompromised patients. These proteins have been characterized to better understand their specific role in infection and exploit their capacity to elicit an immune response. 

Other targeted molecules, such as the penicillin-binding protein and proteins of the type III secretion systems, have been evaluated in silico with the aim to combine these proteins with lipopolysaccharides to achieve enhanced immunity against the microorganism, other than the linker and adjuvants typically used. Among these, the type III secretion system has been evaluated as one of the most immunogenic, since it allows the production of substantial amounts of antibodies during the induced immune response [70,71].

## 7. *Staphylococcus aureus*

Another opportunistic pathogen involved in a number of infectious conditions is *Staphylococcus aureus* (SA) [72]. SA is becoming increasingly dangerous for its ability to rapidly develop antibiotic resistance and to acquire virulence-factors encoding genes from the environment [73]. SA is a Gram-positive bacterium, belonging to the ESKAPE family, and is also responsible for persistent lung infections that affect pwCF. *S. aureus* is able to become resistant to many antibiotics, such as the well-known methicillin-resistant *S. aureus* (MRSA), but also the subsequent vancomycin-resistant *S. aureus* (VRSA), which is why new treatments different from antibiotics are needed [72,73]. As vaccines developed against *S. aureus* have not shown effective results during clinical trials, the possibility to choose among different antigens, facilitated by computational analysis and the availability of the genomes, could improve the identification of new and better targets [68]. As in the case of *P. aeruginosa*, a chimeric vaccine could be very effective against SA. Moreover, a vaccine with a different number of antigens and epitopes could be developed to target both the virulence factors and the bacterial surface elements. This strategy leads to avoiding possible cross-reactions or toxicity with the human host [73]. 

For the development of a multi-epitope vaccine (MEV) against *S. aureus*, several membrane and extracellular proteins, belonging to the virulence factors and are essential for adhesion to the host cells, have been taken into consideration [72]. A small number of these proteins, such as secretory antigens and binding proteins, have been elected for further analysis of the related epitopes to identify the best putative vaccine candidates. The toxicity, immunogenicity and the frequencies of the different class of epitope in *S. aureus*, helps to discriminate among all the possible epitopes, in order to choose the best ones. Furthermore, a future candidate vaccine needs linkers for the epitopes to improve functionality, stability and expression. Moreover, the chosen antigenic proteins must be different from every human protein to avoid cross-reaction, and it could also be characterized by a significant size, since previous studies confirmed that high-length molecules maintain their stability [70]. 

Membrane-surface exposed or secreted proteins have been evaluated as future vaccines against *S. aureus*. In particular, the surface-protein, foldase PrsA, was found to be highly antigenic due to its role in virulence processes and adhesion. Also, it is involved in the formation of the microbial cell wall, making it a good candidate for vaccine development. EssA, instead, is a well conserved ESAT-6 machinery protein implicated in the secretion of proteins essential in the pathogenicity events [73]. Finally, several proteins involved in adhesion and being part of the polysaccharides capsule have been identified by RV, indicating the possibility to develop different MEVs. 

Among the virulence factors, *S. aureus* hyaluronate lyase has been chosen as a putative vaccine candidate, as it is essential for pathogenesis [74]. Besides hyaluronidases, Staphpain is also an interesting virulence factor involved in the erosion of the host connective tissues, in biofilm formation and protection against the immune responses [69]. Both are examples of biofilm-associated proteins which can be exploited as immunogenic targets in the development of MEV. 

Another example is the Staphilokinase, essential for the penetration of the bacterium into the cell, that forms a complex with host proteins, thus making the microorganism resistant against the immune response. In this case, several epitopes have been identified through RV among different antigenic players, and their IC_50_ were evaluated and filtered to keep the epitopes with the best immune response [74]. Staphilokinase, as well as other bacterial antigenic proteins identified by bioinformatic approaches, have been defined by their protective signatures for the pathogen to immune response; this group of proteins is named “protectome”. Even if their mechanism of action against the immune system is not known yet, they can become an interesting target for the development of vaccines. Indeed, the idea is to target virulence factors of *S. aureus*, which can impair the bacterium; therefore, these proteins must be both immunogenic and be part of biological pathways [75]. In this sense, RV allows the mapping of all the epitopes which create an immune response. For example, a series of epitopes have been identified from *S. aureus* superantigens, which anomalously activate the T-cells and, consequently, the immune system. Considering the superantigens, a MEV can be developed from glycosylation and phosphorylation sites, which allows them to achieve a better antigenicity and thus a more powerful vaccine [76]. Despite this, there is still an urgent need to define effective epitopes in the attempts to develop new vaccines, also taking advantage of previous failures, mostly due to the bacterium intricate genome.

## 8. Conclusions

The development of RV over the last 20 years, thanks to the implementation of high-throughput bioinformatics methods and different software, has resulted in effective vaccines against different pathogens. Indeed, VaxiJen, Jenner-Predict, NERVE and VacSol are nowadays commonly used to select the best antigens among the whole pathogens’ genome as putative vaccine candidates.

However, extensive work still needs to be conducted, for example, against the five major bacteria involved in infections of individuals with CF disease. *Burkholderia cepacia*, *Haemophilus influenzae*, *Mycobacterium abscessus*, *Pseudomonas aeruginosa* and *Staphylococcus aureus* infections are hard to eradicate with actual therapeutic regimens that are mostly based on antibiotic administration. In this perspective, prevention remains the best strategy for improving patients with cystic fibrosis’ health and lifestyle, which is why many efforts have moved towards the discovery of new vaccines.

RV has been shown to be faster and more efficient than traditional approaches in identifying novel epitopes, partly due to major advances in the development of new software and methods for whole-genome sequencing. For this reason, several researchers have recently undertaken studies along these lines for CF pathogens as well. At this stage, the obtained beneficial discoveries are related mostly to in silico predictions, and further validation of epitopes is necessary in both preclinical and clinical phases in order to assess their capability to stimulate the immune response. Indeed, a number of putative targets have been identified and many epitopes have been evaluated, allowing the design of several putative vaccines, including multi-epitope vaccines that could provide protection against multiple infections: a frequent situation in pwCF. These important advancements pave the way for the next steps towards validation of these putative vaccines, through in vitro and in vivo experiments, up to clinical trials.

Given the scientific soundness and the positive preliminary data on vaccines designed by reverse vaccinology, we could expect positive results in developing effective vaccines that could protect pwCF from infections of the main five pathogens, possibly for their entire lives.

## Data Availability

Not applicable.
